# Assessment of acyl-CoA cholesterol acyltransferase (ACAT-1) role in ovarian cancer progression—An *in vitro* study

**DOI:** 10.1371/journal.pone.0228024

**Published:** 2020-01-24

**Authors:** Vijayalakshmi N. Ayyagari, Xinjia Wang, Paula L. Diaz-Sylvester, Kathleen Groesch, Laurent Brard

**Affiliations:** 1 Division of Gynecologic Oncology, Department of Obstetrics and Gynecology, Southern Illinois University School of Medicine, Springfield, IL, United States America; 2 Center for Clinical Research, Southern Illinois University School of Medicine, Springfield, IL, United States America; 3 Simmons Cancer Institute at Southern Illinois University School of Medicine, Springfield, IL, United States America; Duke University School of Medicine, UNITED STATES

## Abstract

Abnormal accumulation of acyl-CoA cholesterol acyltransferase-1 (ACAT-1) mediated cholesterol ester has been shown to contribute to cancer progression in various cancers including leukemia, glioma, breast, pancreatic and prostate cancers. However, the significance of ACAT-1 and cholesterol esters (CE) is relatively understudied in ovarian cancer. In this *in vitro* study, we assessed the expression and contribution of ACAT-1 in ovarian cancer progression. We observed a significant increase in the expression of ACAT-1 and CE levels in a panel of ovarian cancer cell lines (OC-314, SKOV-3 and IGROV-1) compared to primary ovarian epithelial cells (normal controls). To confirm the tumor promoting capacity of ACAT-1, we inhibited ACAT-1 expression and activity by treating our cell lines with an ACAT inhibitor, avasimibe, or by stable transfection with ACAT-1 specific short hairpin RNA (shRNA). We observed significant suppression of cell proliferation, migration and invasion in ACAT-1 knockdown ovarian cancer cell lines compared to their respective controls (cell lines transfected with scrambled shRNA). ACAT-1 inhibition enhanced apoptosis with a concurrent increase in caspases 3/7 activity and decreased mitochondrial membrane potential. Increased generation of reactive oxygen species (ROS) coupled with increased expression of p53 may be the mechanism(s) underlying pro-apoptotic action of ACAT-1 inhibition. Additionally, ACAT-1 inhibited ovarian cancer cell lines displayed enhanced chemosensitivity to cisplatin treatment. These results suggest ACAT-1 may be a potential new target for the treatment of ovarian cancer.

## Introduction

Epithelial ovarian cancer has the highest mortality rate among all gynecologic cancers with no curative treatment and poor survival [1, 2]. Although most ovarian cancer patients respond to initial cytoreductive surgery followed by standard chemotherapy, the majority will experience disease recurrence [2–6]. Given the poor response to current second-line or third-line chemotherapy drugs, there is a critical need for developing personalized and targeted treatment strategies based on highly reliable predictive and prognostic biomarkers.

Numerous studies are being carried out to decode the altered lipid metabolic profiles of cancer cells to formulate cancer specific therapeutic strategies. Altered lipid metabolism leads to increased cancer cell proliferation, migration and invasion resulting in metastasis [7–9]. Identification of mediators aiding these processes is essential for developing therapies to target cancer metastasis. Altered lipid metabolism involves increased expression of both lipogenic and lipolytic enzymes to store and utilize newly synthesized lipids. Excessive lipids and cholesterol in cancer cells are converted to triglycerides and cholesteryl esters (CE) for storage in lipid droplets (LDs). Several reports indicate increased amount of lipid droplets in various types of tumors including leukemia, glioblastoma, renal clear cell carcinoma, and cancers of the prostate, colon, breast and pancreas [10–16]. As observed in these cancers, CE were shown to be the major component of LDs within cancerous tissue as compared to normal tissue [17]. Increased levels of CE were shown to promote tumor proliferation, invasiveness and survival via reduced *de novo* lipid synthesis, inducing lipid raft formation and finally altering cell signaling [18–20]. Lowering levels of CE was found to inhibit cell proliferation in breast cancer [10] lymphocytic leukemia [11] and glioblastoma [12] cell lines *in vitro*. These observations suggest that strategies targeting the prevention of CE accumulation may prove beneficial for treatment of ovarian cancer.

Free excess cholesterol is esterified to CE by acyl-CoA cholesterol acyltransferase (ACAT) [21] which exists in two isoforms, ACAT-1 and ACAT-2. Whereas ACAT-2 is associated with lipoprotein particle secretion, ACAT-1 is ubiquitous and known to maintain cellular cholesterol homeostasis. ACAT-1 mediated accumulation of CE was shown to positively correlate with poor survival in pancreatic and prostate cancers [12, 13, 22–27]. While the role of ACAT-1/CE accumulation is being studied in various cancers, information regarding the contribution of these mediators in ovarian cancer is relatively scarce. In this *in vitro* study, we determined the expression levels and contribution of ACAT-1 in ovarian cancer progression utilizing a panel of ovarian cancer cell lines. The role of ACAT-1 in tumor cell aggression was studied by blocking ACAT-1 expression/activity in OC-314, SKOV-3 and IGROV-1 cell lines using ACAT-1 specific short hairpin RNA (shRNA). Important tumor associated activities, such as cell migration, invasion and proliferation capabilities, were compared between ACAT-1 inhibited cell lines and their respective scrambled control cell lines. Furthermore, to investigate the molecular mechanism(s) underlying ACAT-1 mediated cancer progression, we studied the effect of ACAT-1 inhibition on cell cycle, apoptosis and mitochondrial membrane potential. Additionally, we evaluated the possible involvement of reactive oxygen species (ROS) and tumor suppressor p53 in ACAT- 1 mediated effects. Finally, we studied the effect of ACAT-1 inhibition on chemosensitivity towards cisplatin as previous reports have linked cholesterol/CE to drug resistance [28, 29].

## Materials & methods

### Cell lines and chemicals

Human primary ovarian epithelial cells (H-6036) were obtained from Cell Biologics, (Chicago, IL, USA). Human ovarian carcinoma cell lines, OC-314 and SKOV-3 were obtained from Dr. McAsey’s laboratory (Department of Obstetrics & Gynecology, SIU School of Medicine, Springfield, IL). Isogenic ovarian cancer cell line pairs, e.g., A2780 / A2780-CDDP and IGROV-1 / IGROV-1CDDP were obtained from Dr. Brodsky (Brown University, Providence, RI). As previously reported [30], all cell lines were maintained in DMEM media (Sigma) supplemented with 10% heat inactivated FBS (Hyclone), 10 mM HEPES (Mediatech), 4 mM L-glutamine (Mediatech), 1 mM sodium pyruvate (Mediatech), 1X non-essential amino acids (Mediatech), 100 IU penicillin (Mediatech) and 100 μg/ml streptomycin (Mediatech). All cell lines were cultured at 37°C in a humidified atmosphere with 5% CO_2_. SKOV-3, IGROV-1 and OC314 cell lines were authenticated by the ATCC using STR profiling technique. All cells tested negative for mycoplasma. Avasimibe used in the experiments was purchased from Selleckchem, TX, USA.

Antibodies for ACAT-1, ACAT-2 and Phosphatase and tensin homolog (PTEN) were obtained from abcam (Cambridge, MA, USA). Antibodies for p53, p21, p27, Bax, bcl-2 and β-actin were purchased from Cell Signaling Technology, Inc (Danvers, MA, USA).

### Quantitative real time-PCR (qRT-PCR) for ACAT-1 mRNA expression

Total RNA was extracted from cells using RNeasy mini-kit (Qiagen, Hilden, Germany) following manufacturer’s instructions. After RNA yield and quality were assessed, samples were stored at −80°C until use. 50 ng of extracted RNA was used to perform qRT-PCR analysis of ACAT-1 gene expression using Invitrogen^™^ Ambion^™^ Cells-to-CT^™^ Power S 1-Step Power SYBR Green Kit. The kit’s instructions were followed with the exception that extracted RNA was used instead of cells directly. The ACAT-1 specific primers were purchased from Integrated DNA Technologies, Inc. (Coralville, Iowa, USA). The sequences of primers used for ACAT-1 qRT-PCR are listed as below:

Forward: forward primer, 5′-CAAGGCGCTCTCTCTTAGATGAAC-3′;

Reverse: reverse primer, 5′-GATAAAGAGAATGAGGAGGGCAATAA-3′

*18srRNA* was used as a reference housekeeping gene. The threshold cycle (Ct) values were normalized to the housekeeping gene to quantify ACAT-1 mRNA (Comparative Ct method). Data were expressed as mean ± SD of triplicate experiments.

### Western blot analysis

Western blotting was performed to evaluate expression of ACAT-1, ACAT-2 and other important regulators of cell cycle and apoptosis. Cell seeding, cell lysis and western blotting were done as described previously with slight modifications [30]. In brief, cells were harvested and lysed in RIPA lysis buffer (1% Triton X-100, 150 mM NaCl, 1mM EGTA, 50 mM Tris-HCl, 0.1% sodium dodecyl sulfate (SDS), 1 mM PMSF and 1X complete Protease inhibitor (A32955); ThermoFisher Scientific, MO, USA) and protein concentration was determined using a bicinchoninic acid (BCA) protein assay reagent kit (Bio-Rad, USA). Cell lysates were subjected to western blotting. After overnight incubation with the respective primary antibodies at 4°C and subsequent incubation with the appropriate secondary antibodies (Licor), the proteins on the blots were detected using a Licor image analyzer. Primary antibodies were diluted as follows: Anti-ACAT1 (1:500), Anti-ACAT2 (1:500), p53 (1:1,000), anti-PTEN (1:1,000), anti-p21 (1:500), anti-p27 (1:1000), anti-Bax (1:1,000), anti-bcl2 (1:500) and anti-β-actin (1:1,000).

### Quantification of ACAT-1 by ELISA

ACAT-1 protein from cell supernatants was quantified using the ACAT-1 ELISA Kit (Human) from Aviva Systems Biology (San Diego, CA, USA). Briefly, 4 × 10^5^ cells were seeded into a six well plate and incubated for 48 hours at 37°C in a humidified atmosphere with 5% CO_2_. After the stipulated time, media were collected from each well and centrifuged to remove any particulates. The assay was performed immediately with the supernatant according to the kits instructions. The absorbance was measured at 450 nm using synergy H1 microplate reader (BioTek, VT, USA). Data were expressed as mean ± SD of triplicate experiments. Ovarian cancer cell lines were compared against primary ovarian epithelial cells (normal controls).

### Immunocytochemical staining

Cells grown on glass coverslips were fixed with 4% paraformaldehyde and permeabilized using 0.1% Triton X-100 in PBS. Cells were subsequently incubated overnight with ACAT-1 primary antibody (1:50 dilution) at 4°C, probed with FITC labeled secondary antibody and observed under Olympus DP73 fluorescence microscope. Cell were counterstained with DAPI for nuclei staining (blue).

### Quantitative analysis of CE, free cholesterol and total cholesterol from ovarian cancer cell lines

Quantification of CE from ovarian cancer/normal cells was done using Total Cholesterol and Cholesteryl Ester Colorimetric Assay Kit (Biovision; Milpitas, CA, USA) following the manufacturer’s instructions. This kit detects total cholesterol (cholesterol and CE) in the presence of cholesterol esterase in the reaction. Briefly, 1 × 10^6^ cells were processed for lipid extraction by adding 200 μL of chloroform: Isopropanol: NP-40 (7:11:0.1) in a micro homogenizer and centrifuged at 15,000 x g. All the liquid phase (organic phase) was transferred to a new tube and air dried at 50ºC to remove the chloroform. The samples were kept under vacuum to remove organic traces. The dried samples were then dissolved in 200 μL of cholesterol assay buffer by sonication and vortexing until homogeneous. 50 μL of the sample was used for assay of CE, free cholesterol (FC) and total cholesterol (TC) concentrations. Data was expressed as mean ± SD of against primary ovarian epithelial cells (normal controls).

### Cell viability assay

Cell viability was determined by Presto Blue cell viability reagent (Invitrogen, CA, USA) as described previously [30]. In brief, ovarian cancer cell lines were plated into 96-well plates (5000 cells/well) and incubated for the required time intervals. A minimum of 2–4 hours prior to the end of incubation time, Presto Blue reagent was added followed by measurement of fluorescence (540 nm excitation/590 nm emission). All test cells were compared against their respective control cells (considered as 100% viable). Data were expressed as mean ± SD of triplicate experiments.

For assessment of cisplatin IC_50_ values, cells plated in 96-well plates (5000 cells/well) overnight were treated with cisplatin at concentrations ranging from 1.57–400 μM for a total of 48 hours and cell viability was assessed as described above. IC_50_ value was obtained by fitting the data with a sigmoidal dose response model using GraphPad software. Data were expressed as mean ± SD of triplicate experiments.

### Stable transfection for ACAT-1 gene knock-down

Stable ACAT-1 knockdown was done using ACAT-1 shRNA lentiviral particle purchased from Santa Cruz Biotechnology, Inc. (Dallas, TX, USA). ACAT-1 specific shRNA was transfected into the cells (OC-314, SKOV-3 and IGROV-1) according to the manufacturer’s protocols using polybrene transfection reagent. Stable transfected cells were selected under 2 μg/mL puromycin treatment. To assess nonspecific effects of transfection, cells were transfected with scrambled shRNA lentiviral particles (Santa Cruz, sc-108080) and are referred to as “scrambled controls” for comparative purposes.

### Cell proliferation assays

Cell proliferation studies were performed using CyQuant cell proliferation assay kit (ThermoFisher Scientific, NY, USA) which accurately estimates cell number based on the DNA content. Cells were seeded into a 96-well plate at a density of 1000 cells / 100 μL per well and incubated for 1, 2, 3, 4, 6, and 14 days. At the end of each time point, 100 μL CyQuant solution was added to the wells and fluorescence was measured (excitation 485/emission 530 nm) using synergy H1 microplate reader (BioTek, VT, USA). ACAT-1 inhibited cell lines were compared against their respective scrambled controls for each cell line. Data were expressed as mean ± SD of triplicate experiments.

### Colony formation assay

Colony formation assay was performed following a standard protocol [31]. In brief, 200 cells were seeded in 6-well plates and incubated for 21 to 28 days to see difference in number of colonies. Media changes were done regularly to maintain cell health. At the end of incubation, surviving colonies were fixed with 90% ethanol, stained with crystal violet and counted by using ImageJ software. Data were expressed as mean ± SD of triplicate experiments.

### Migration and invasion assays

Migration and Invasion assays were performed using FluoroBlok^™^ cell culture inserts with 8 μm pore-size PET membrane. Cell culture inserts with BD matrigel coated PET membrane were used for invasion assays whereas uncoated inserts were used for migration assays. Briefly, 1 ×10^5^ cells were seeded in the upper chamber of the trans-wells in 0.5 ml serum-free media. The lower chamber contains 750 μL of media with 20% FBS. Cells were allowed to migrate for 22 hours at 37°C, 5% CO_2._ Quantitation of cell migration and invasion was done by labeling the cells with a fluorescent dye calcein AM and measuring the fluorescence of migrated/invaded cells using a synergy H1 microplate reader (BioTek). As BD FluoroBlok membrane blocks the light from 490–700 nm with >99% efficiency, only migrated/invaded cells are detected by the bottom-reading fluorescence plate reader. Data were expressed as mean ± SD of triplicate experiments.

### Cell-cycle analysis

Cell cycle analysis was carried out using Muse^®^ Cell Cycle Assay Kit (MCH100106) following the manufacturer’s instructions. Briefly, 70% ethanol fixed cells were washed and stained with a pre-mixed reagent containing nuclear DNA stain propidium iodide and RNAse A. Then, cells were categorized using Muse cell analyzer (MilliporeSigma, CA, USA). The Muse Cell Cycle Software module performs calculations automatically and categorizes the cells into different cell cycle phases based on propidium staining of nuclei.

### Morphological studies to detect apoptosis

Nuclear condensation indicative of apoptosis was assessed using NucBlue Live Cell Stain Hoechst 33342 (Invitrogen, Carlsbad, CA). This qualitative test was performed as described previously [30, 32]. In brief, cells (1 × 10^5^ cells) were seeded in 60 mm^2^ culture dishes and incubated for 4 days. At the end of the stipulated time, cells were washed and stained with Hoechst stain (2 drops/mL of media) for 15 minutes at 25°C and observed under a fluorescent microscope. Representative images were taken with an inverted microscope (Olympus H4-100, CCD camera) and 20× objective. Further quantification of apoptosis was done using Muse’s Annexin V & Dead Cell Assay Kit (MCH100105, MilliporeSigma, CA, USA) following the manufacturer’s instructions. Briefly, cells were incubated with Muse Annexin V & Dead Cell reagent at room temperature in the dark and analyzed using Muse® Cell Analyzer (Merck Millipore, Burlington, MA, USA). The Muse Software Module performs calculations automatically.

### Mitochondrial transmembrane potential assay

Mitochondrial transmembrane potential was determined Muse’s MitoPotential Assay Kit (MCH100110, MilliporeSigma, CA, USA), which simultaneously measures changes in mitochondrial potential (early hallmark of apoptosis) and cell death using MitoPotential Dye and 7-AAD. Ovarian cancer cell lines (1 × 10^6^) were seeded into a 100 mm^2^ culture dishes and incubated for 3 days. Cells were harvested, and stained with Muse^™^ MitoPotential Dye for 30 min at 37°C followed by addition of 7-AAD. The Data was acquired using Muse® Cell Analyzer (Merck Millipore, Burlington, MA, USA). The Muse Software Module performs calculations automatically.

### Caspase 3/7 assay

Caspase 3/7 activity was measured using Caspase-Glo 3/7 assay kit (Promega, Madison, WI, USA) following the manufacturer's recommendations. Briefly, 1 × 10^4^ cells were plated per well on a 96-well plate and incubated for 48 hrs. Caspase-Glo 3/7 reagent was added and incubated for 30 min at room temperature. The luminescence intensity was measured using luminoskan (ThermoScientifics, NY, USA). Data were expressed as mean ± SD of triplicate experiments.

### Estimation of reactive oxygen species (ROS) production

ROS were detected via carboxy-H2DCFDA using flow cytometry as described previously [30]. Briefly, 1 × 10^6^ cells were seeded in a petri dish and incubated for 3 days after which the cells were washed and re-suspended in fresh PBS and incubated with 5 μM 5,6-carboxy-2′,7′-dichlorodihydrofluorescein diacetate (carboxy-H2DCFDA, C400, Invitrogen, Eugene, Oregon, USA) for 30 min at 37°C. The cells were washed twice with DPBS, re-suspended in an equal volume of DPBS and fluorescence measured with flow cytometry. Data was acquired on a BD Accuri C6 flow cytometer and analyzed using Accuri C6 software (BD Immunocytometry-Systems, San Jose, CA). Twenty thousand cells were analyzed for each sample.

### Statistical analysis

All experiments were performed at least in triplicate. Data are expressed as the means ± standard deviation (SD). Statistical analyses were performed using Student’s t-test. Differences were considered statistically significant at P<0.05.

## Results

### Increased expression of ACAT-1 in ovarian cancer cell lines

**Fig 1:**
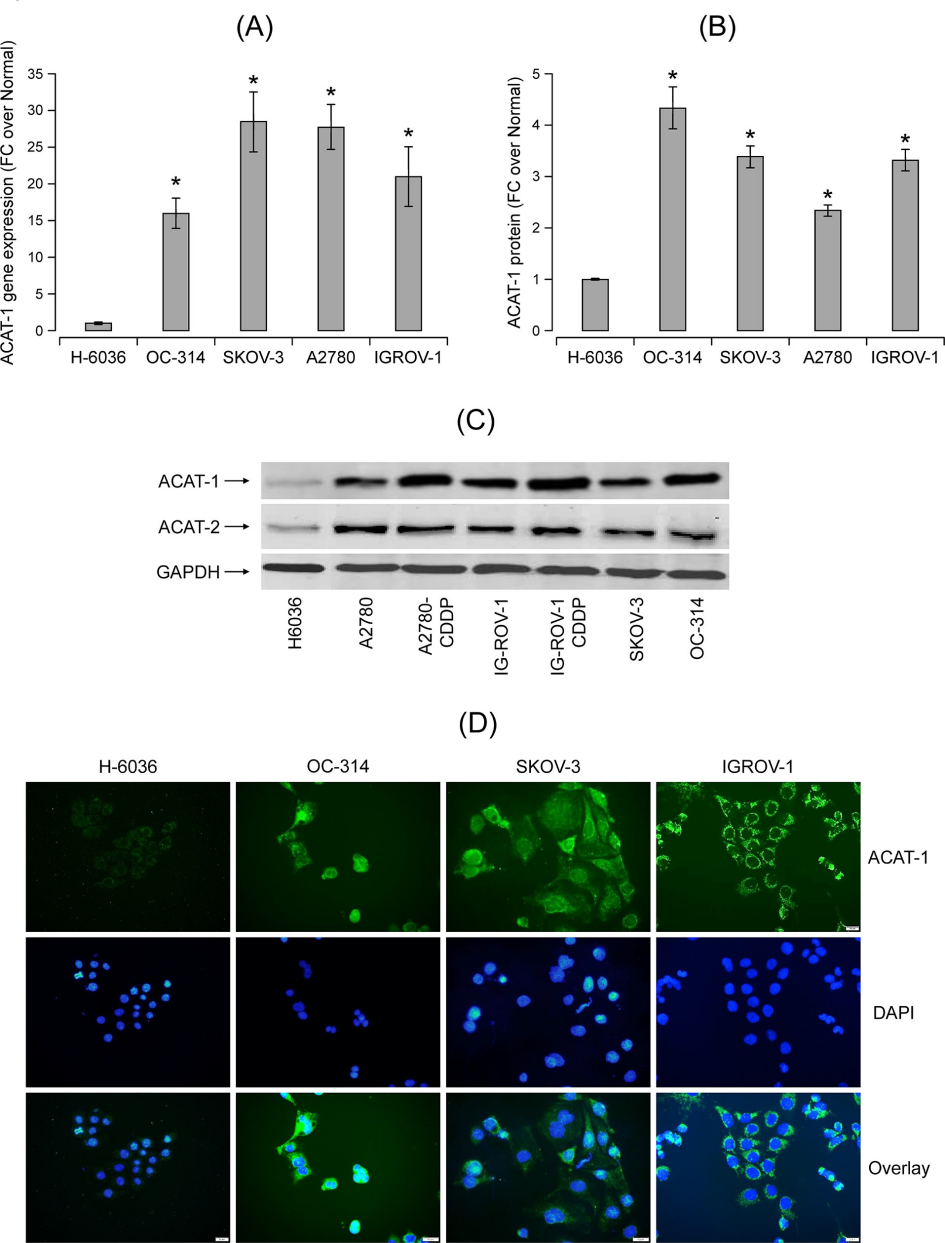
ACAT-1 expression and CE levels in a panel of ovarian cancer cell lines (OC-314, SKOV-3, A2780, IGROV-1) in comparison to primary ovarian epithelial cells, H-6036 (normal controls) **(A)** qRT-PCR analysis of ACAT-1 mRNA expression in a panel of ovarian cell lines. The threshold cycle (Ct) values were normalized to the housekeeping gene 18srRNA to quantify ACAT-1 mRNA (Comparative Ct method). Data represent mean fold change (FC) of ACAT-1 mRNA expression in cancer cell lines over normal control cells (H-6036), Mean FC ± SD of triplicate experiments, *P < 0.05 versus normal control cells, Student’s t-test. **(B)** ACAT-1 protein levels quantified by ELISA. Data represent mean FC ± SD of triplicate experiments. *P < 0.05 versus normal control cells, Student’s t-test. **(C)** Western blot analysis of the ACAT-1 and ACAT-2 proteins in a panel of ovarian cell lines. GAPDH is used as the loading control. **(D)** Immunocytochemical staining for ACAT-1 in various ovarian cancer cell lines using primary antibody against ACAT-1 with FITC labeled secondary antibody (green) and DAPI counterstain (blue). Representative images were taken with an inverted microscope (Olympus H4-100, CCD camera) and a 40X objective.

Using qRT-PCR, we found significant expression of ACAT-1 mRNA in a panel of ovarian cancer cell lines compared to primary ovarian epithelial cells, H-6036 (normal controls) (Fig 1A). Significantly higher expression of ACAT-1 protein in cancer cell lines compared to normal controls was confirmed using ELISA (Fig 1B).

Since ACAT exists predominantly in two isoforms (ACAT-1 and ACAT-2), it is essential to understand the expression levels of both isoforms in ovarian cancer cell lines. Western blot analysis show significant expression of ACAT-1 protein in cancer cell lines compared to normal control cells (Fig 1C). This result correlates with qRT-PCR and ELISA data. Interestingly, cisplatin-resistant variants (A2780-CDDP and IGROV1-CDDP) of isogenic cell line pairs showed higher ACAT-1 expression compared to their respective cisplatin-sensitive counterparts (A2780 and IGROV-1). ACAT-2 expression was observed in all ovarian cell lines however the difference in expression levels between cancer cells and normal control cells is relatively less when compared to ACAT-1 (Fig 1C). Immunostaining data further confirmed the enhanced expression of ACAT-1 protein in ovarian cancer cell lines versus normal control cells (Fig 1D). Therefore, we focused on ACAT-1 to further understand the role of ACAT in ovarian cancer progression.

### Increased cholesterol ester levels in ovarian cancer cell lines

**Fig 2:**
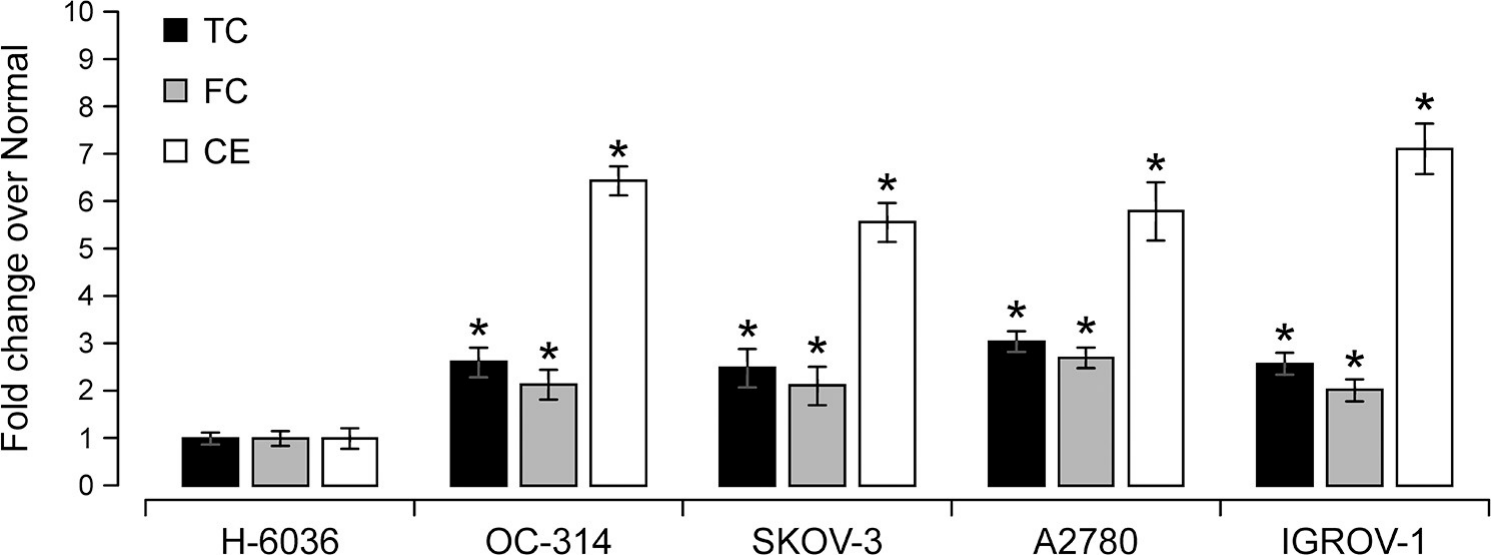
Comparison of fold changes of total cholesterol (TC), free cholesterol (FC) and cholesterol ester (CE) levels between normal control cells and a panel of ovarian cancer cell lines. Values represent mean FC ± SD of triplicate experiments, *P < 0.05 versus normal control cells, Student‘s t-test.

ACAT-1 mediates esterification of cholesterol to CE for its storage in LDs; thus, we assessed the levels of TC, FC and CE in ovarian cancer cell lines. In positive correlation with ACAT-1 data, CE levels in ovarian cancer cell lines were significantly higher (5–7 fold) than normal control cells (Fig 2). Besides CE, a significant increase in TC (2–3 fold) and FC levels (2–3 fold) was observed in cancer cell lines compared to normal control cells. All together, these results confirm increased lipid content in cancer cell lines. Therefore, any strategies targeting cancer specific lipid profiles appear promising for further research.

### ACAT inhibition reduced cell viability in ovarian cancer cell lines

**Fig 3:**
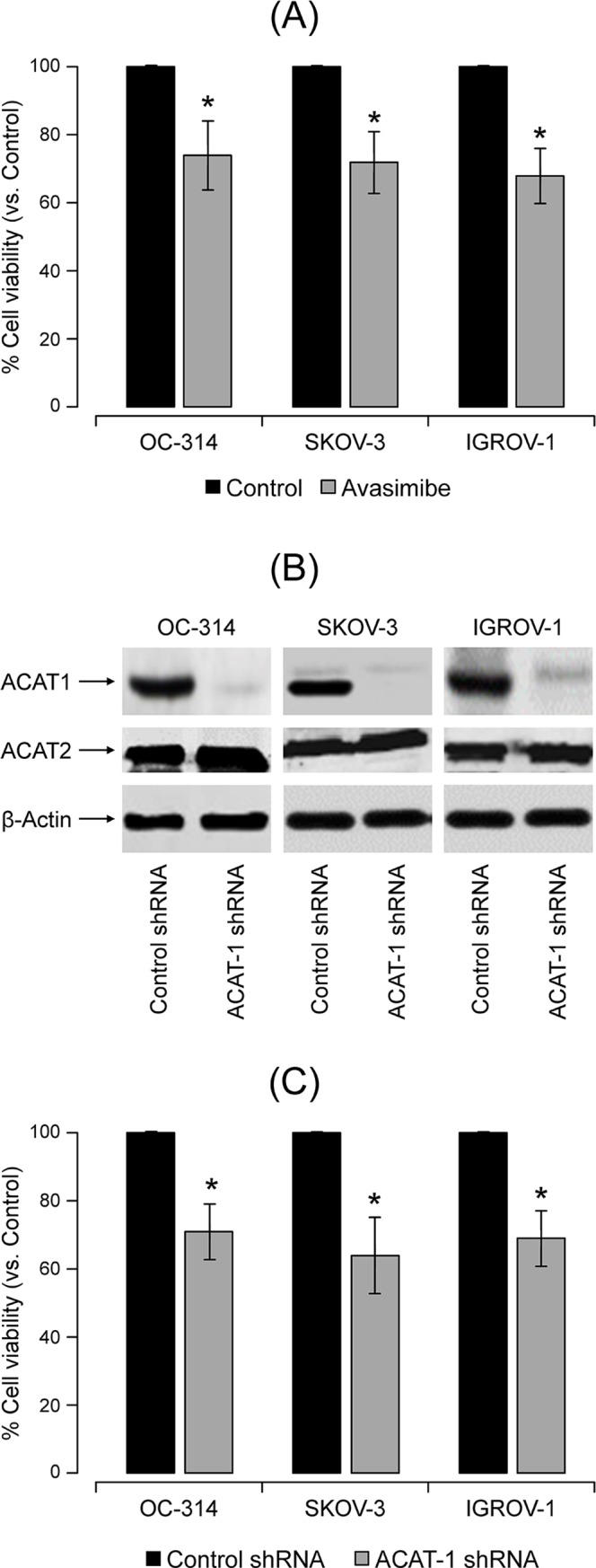
Effect of ACAT-1 inhibition on viability of a panel of ovarian cancer cell lines. **(A)** Cell viability assay of a panel of ovarian cancer cell lines treated with ACAT inhibitor avasimibe for 3 days. Vehicle treated cells (media with 1% DMSO) were considered as control against which treated cells were compared. Data expressed as mean ± SD of triplicate experiments. *P < 0.05, compared to control, Student’s t-test. **(B)** Confirmation of efficient stable ACAT-1 knock down assessed by western blot. β-actin was used as the loading control. **(C)** Assessment of cell viability upon stable knock-down of ACAT-1, in a panel of ovarian cancer cell lines. Data are expressed as mean ± SD of triplicate experiments. *P < 0.05, compared to the respective scrambled controls, Student’s t-test.

To further understand the role of ACAT-1 in tumor progression, we performed cell viability assays using three ovarian cancer cell lines SKOV-3, OC-314 and IGROV-1. Initially, we inhibited ACAT using the non-specific inhibitor, avasimibe. Cells treated with avasimibe for 3 days exhibited significantly reduced cell viability (PrestoBlue assay) compared to untreated controls. As shown in Fig 3A, treatment with 5μM avasimibe for 3 days reduced cell viability by 26 ± 4%, 28 ± 2% and 32 ± 6% in OC-314, SKOV-3, and IGROV-1 cell lines, respectively, compared to their respective untreated controls. Since avasimibe is a nonspecific ACAT inhibitor, it is essential to identify and focus on the ACAT isoform that contributes to tumor promoting effects. In comparison to ACAT-2, ACAT-1 expression varied significantly between ovarian cancer cell lines versus normal control cells. Therefore, we specifically inhibited ACAT-1 in ovarian cancer cell lines by generating stable ACAT-1 knock down cell lines using shRNA sequences corresponding to ACAT-1. Effective knockdown of ACAT-1 was confirmed by western blotting (Fig 3B). ACAT-2 expression was not altered during the transfection implicating ACAT-1 specific knockdown in all ovarian cancer cell lines (Fig 3B).

ACAT-1 inhibition by stable transfection with shRNA resulted in significant decrease in cell viability (29 ± 5%, 36 ± 6% and 31 ± 5%) in OC-314, SKOV-3 and IGROV-1 cell lines, respectively, compared to their respective scrambled controls, when assessed 6 days post-seeding (Fig 3C).

### ACAT-1 inhibition reduced cell proliferation and colony formation

**Fig 4:**
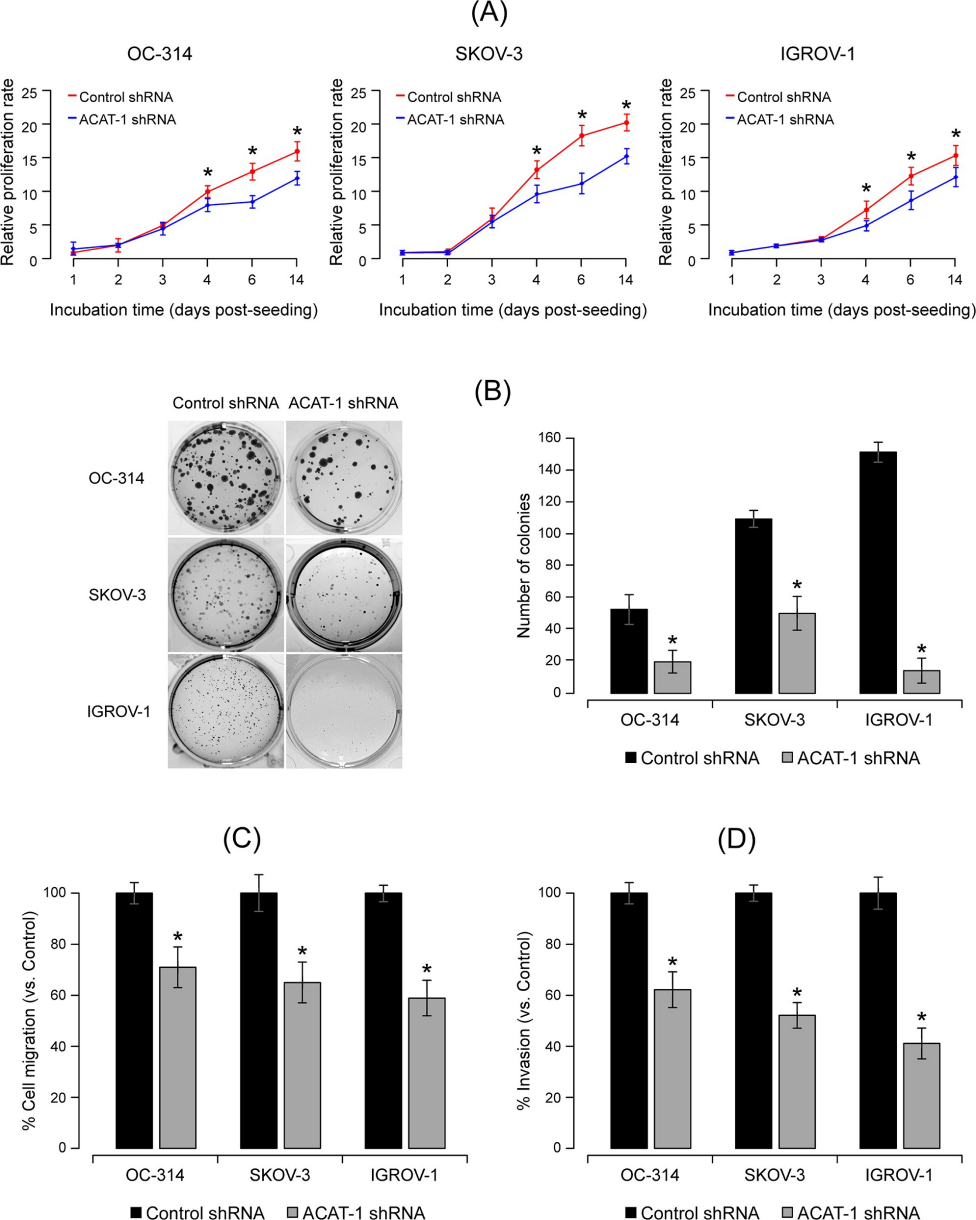
Effect of shRNA mediated ACAT-1 inhibition on ovarian cancer cell proliferation, colony formation, migration and invasion properties **(A)** Cell proliferation assay of ovarian cancer cell lines stably transfected with control shRNA or ACAT-1 specific shRNA at 1, 2, 3, 4, 6, and 14 days post-seeding. Data are shown as mean ± SD of triplicate experiments. *P < 0.05, compared to the respective scrambled controls, Student’s t-test. **(B) Left panel:** Colony formation assay. Cells seeded at 200 cells/100 mm^2^ petri dish were incubated for 21 to 28 days. Surviving colonies were fixed, stained with crystal violet and observed under microscope. **Right panel:** Quantitation of number of colonies. Data were expressed as mean ± SD of triplicate experiments. *P < 0.05, as compared to respective scrambled controls, Student’s t-test **(C)** Cell migration abilities of control shRNA transfected cell lines vs. ACAT-1 shRNA transfected cell lines. **(D)** Cell invasion assay on control sh-RNA vs. ACAT-1 shRNA transfected cells. Data represent mean ± SD of triplicate experiments. *P < 0.05, as compared to respective scrambled controls, Student’s t-test.

To assess the effect of ACAT-1 inhibition on cell proliferation, we performed time course studies ranging from 1 to 14 days post-seeding using CyQuant cell proliferation assay kit that accurately measures cell number based upon the DNA content. As shown in Fig 4A, a significant decrease in the cell proliferation rate was observed in all ACAT-1 knockdown cell lines compared to their respective scrambled controls. Similar to the presto-blue assay, reduced proliferation was observed only from 4 days post-seeding. These results suggest that initially cells may undergo a compensatory mechanism to neutralize the effects of metabolic stress due to ACAT-1 inhibition. However, with progression of time, significant accumulation of metabolic stress may result in decreased cell proliferation.

Unlimited proliferation is the predominant feature of all cancer cells. In order to understand the effect of ACAT-1 inhibition on proliferation, we performed a colony formation assay that measures cells ability to undergo sufficient proliferation to form a colony. Our results clearly show that in all three cell lines, ACAT-1 inhibited cells formed less number of colonies compared to their respective scrambled controls (Fig 4B). These results further confirm the contribution of ACAT-1 in cancer progression.

### ACAT-1 inhibition decreased cell migration and cell invasion

To evaluate whether ACAT-1 inhibition affects tumor aggressiveness, we measured cell invasion and migration abilities of ACAT-1 inhibited and control cell lines. We observed that both migration and invasion capabilities of OC-314, SKOV-3 and IGROV-1 cell lines were markedly suppressed upon ACAT-1 inhibition. Quantitation of results indicated that ACAT-1 inhibition reduced cell migration by 29 ± 7%, 35 ± 5% and 41 ± 7`% in ACAT-1 inhibited (shRNA) cell lines of OC-314, SKOV-3 and IGROV-1 respectively compared to their respective scrambled controls (Fig 4C). Similarly, cell invasion ability was reduced by 38 ± 8%, 48 ± 9%, and 59 ± 6% in ACAT-1 inhibited (shRNA) cell lines of OC-314, SKOV-3 and IGROV-1 cell lines respectively compared to their respective scrambled controls (Fig 4D). Inhibition of cell migration and invasion upon ACAT-1 inhibition supports its role in tumor aggression.

### Effect of ACAT-1 inhibition on cell cycle

**Fig 5:**
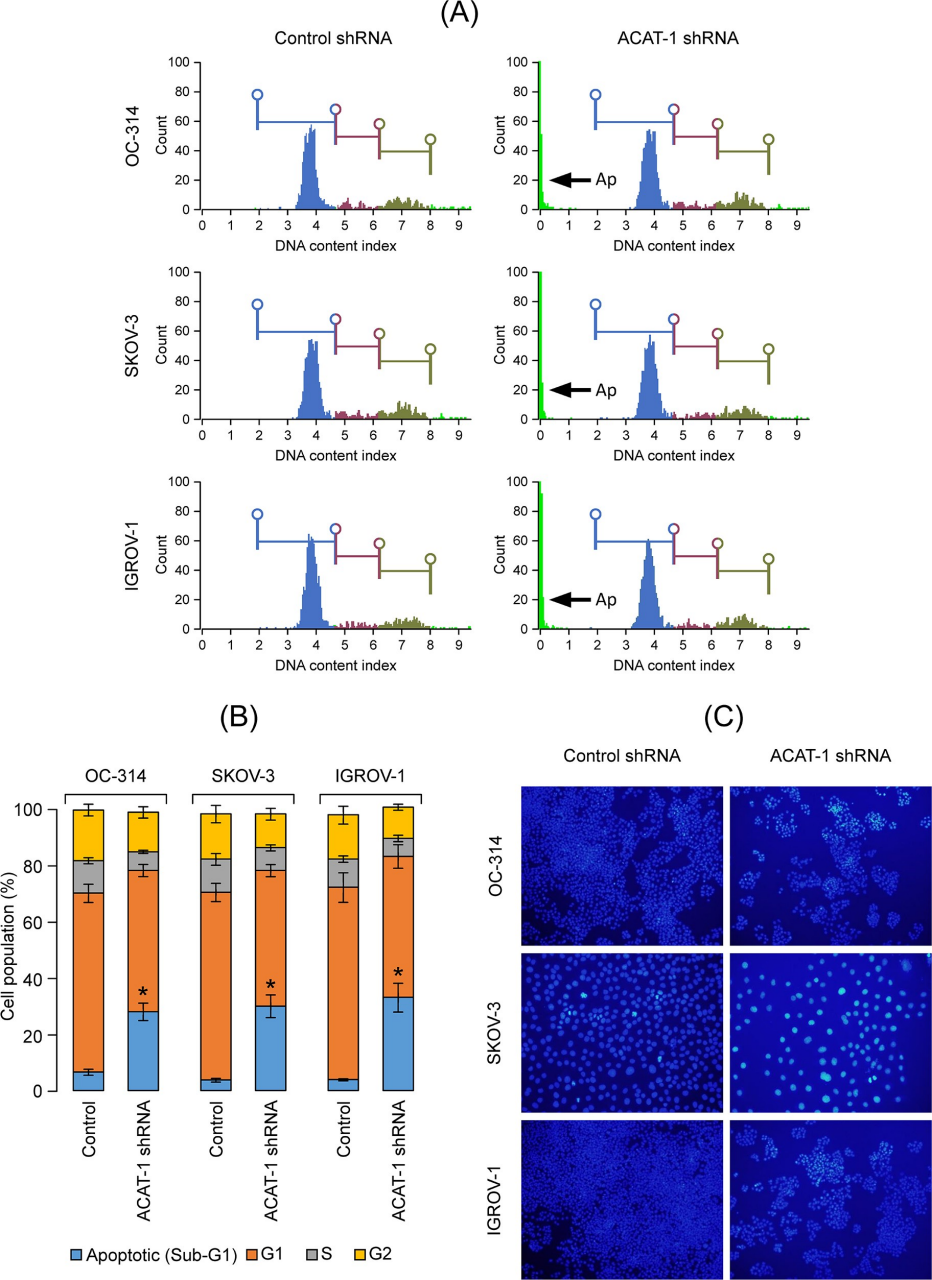
Effect of shRNA mediated ACAT-1 inhibition on cell cycle. **(A):** Cell cycle analysis of ovarian cancer cell lines stably transfected with control shRNA or ACAT-1 shRNA. Arrows (Ap) represent sub-G1 (apoptotic) phase. **(B)**: Graphical representation of cell cycle analysis. Data are presented as mean ± SD of triplicate experiments. *P < 0.05, compared to respective scrambled controls, Student’s t-test. **(C)** Effect of ACAT-1 inhibition on apoptosis. Nuclear condensation indicative of apoptosis was assessed using NucBlue Live Cell Stain Hoechst 33342. Representative images were taken with an inverted microscope (Olympus H4-100, CCD camera) and 20× objective.

In order to understand the mechanisms underlying the suppressive effects of ACAT-1 inhibition on cancer cell growth, we performed cell cycle analysis. Flow cytometry analysis revealed that in comparison to scrambled controls, ACAT-1 inhibited cell lines exhibited a significantly higher number of cells in sub-G1 (apoptotic) phase with the corresponding decrease in G1 phase of cell cycle (Fig 5A). A significant 21 ± 3%, 26 ± 3%, and 29 ± 5% increases in sub-G1 (Ap—apoptotic) phase was observed in OC-314, SKOV-3 and IGROV-1 cell lines, respectively, compared to their respective scrambled controls (Fig 5B).

### ACAT-1 inhibition enhanced apoptosis

**Fig 6:**
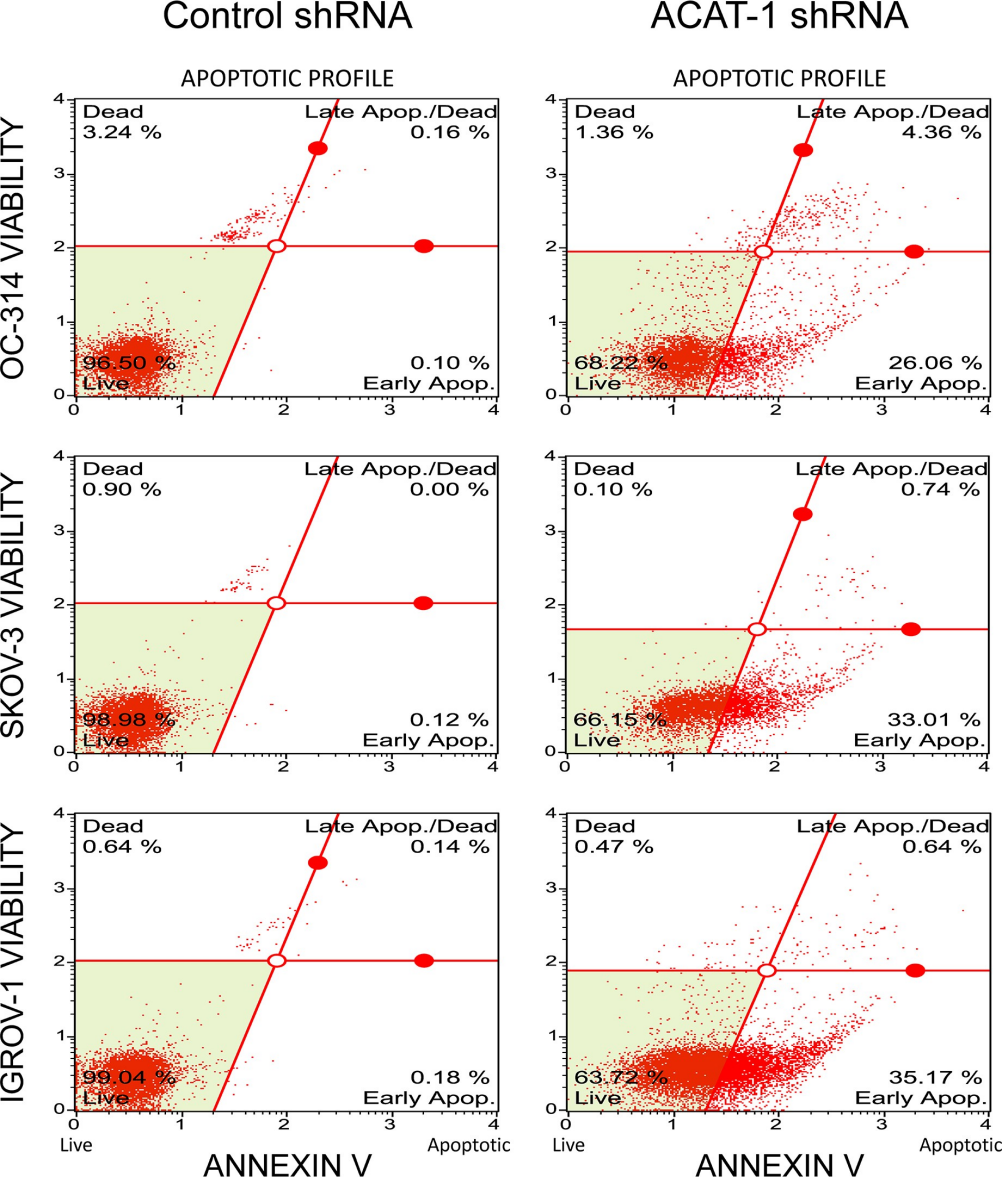
Effect of shRNA mediated ACAT-1 inhibition on apoptosis. Quantification of apoptosis was done using Muse’s Annexin V & Dead Cell Assay Kit. The Muse Software Module performs calculations automatically based on Annexin-V and 7-AAD staining. Data represent mean ± SD of triplicate experiments. *P < 0.05, as compared to respective scrambled control, Student’s t-test.

Since cell cycle data show significant population of cells in sub-G1 (apoptotic) phase in ACAT-1 inhibited cell lines, we performed qualitative assessment of apoptosis using nuclear stain Hoechst 33342. As shown in Fig 5C, Hoechst stained scrambled control cells showed very faint blue fluorescence whereas ACAT-1 inhibited cell lines exhibited stronger blue fluorescence indicative of apoptosis due to highly condensed chromatin. All three cell lines exhibited similar results. Further quantitative assessment of apoptosis was done using Muse’s Annexin V & Dead Cell Assay Kit that detects live, dead, early apoptotic and late apoptotic cells. As shown in Fig 6, ACAT-1 inhibited cell lines OC-314, SKOV-3 and IGROV-1 displayed a 26 ± 3%, 33 ± 4%, and 37 ± 5% increase in early apoptotic cells with corresponding decrease in viable cells (31 ± 5%, 28 ± 3% and 25 ± 2%), respectively, compared to their respective scrambled controls, when assayed at 3 days post-seeding. ACAT-1 inhibition did not cause any significant increase in the number of late apoptotic cells when assayed at 3 days post seeding, compared to their respective scrambled control cell lines. These results consistently indicate growth suppressive effects of ACAT-1 inhibition.

### Mechanisms underlying the suppressive effects of ACAT-1 inhibition on cancer cell growth

In this study, we mainly focused on apoptosis related changes associated with ACAT-1 inhibition to understand the underlying mechanism(s). These include mitochondrial membrane potential change, activities of pro-apoptotic markers such as caspases 3/7 and expression of proteins associated with tumor suppression and ROS generation.

#### ACAT-1 inhibition decreased mitochondrial membrane potential

**Fig 7:**
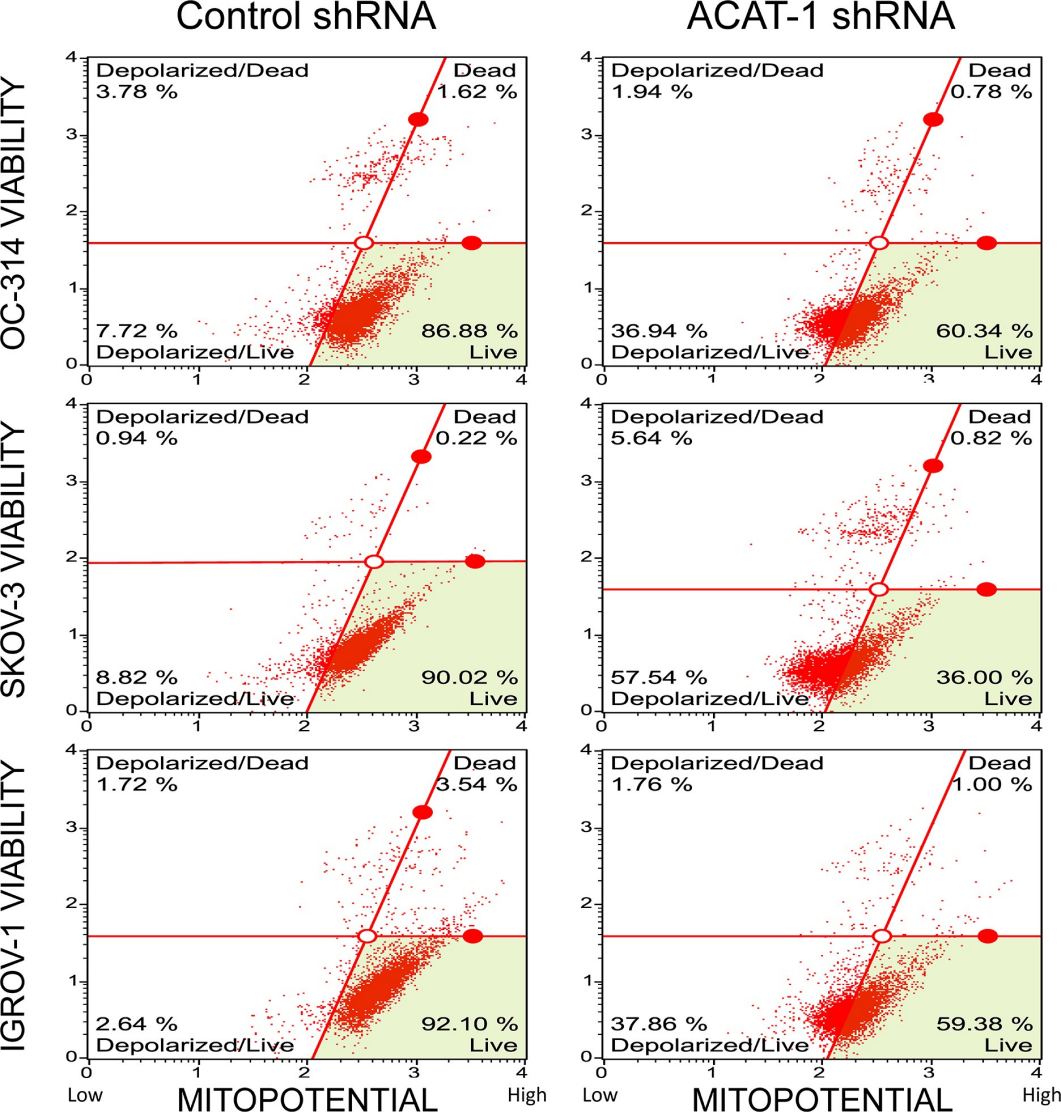
Effect of shRNA mediated ACAT-1 inhibition on mitochondrial transmembrane potential. Detection of changes in mitochondrial trans-membrane potential upon ACAT-1 inhibition using Muse MitoPotential Assay Kit. Data represent mean ± SD of triplicate experiments. *P < 0.05, as compared to scrambled control, Student’s t-test.

Mitochondrial membrane potential changes were assessed using Muse MitoPotential Assay Kit. As shown in Fig 7, the number of depolarized cells increased by 29 ± 4%, 49 ± 8%, and 35 ± 5% in ACAT-1 inhibited cell lines of OC-314, SKOV-3 and IGROV-1, respectively, indicating a decrease in mitochondrial membrane potential compared to the scrambled controls.

#### ACAT-1 inhibition induced caspase3/7 activities

**Fig 8:**
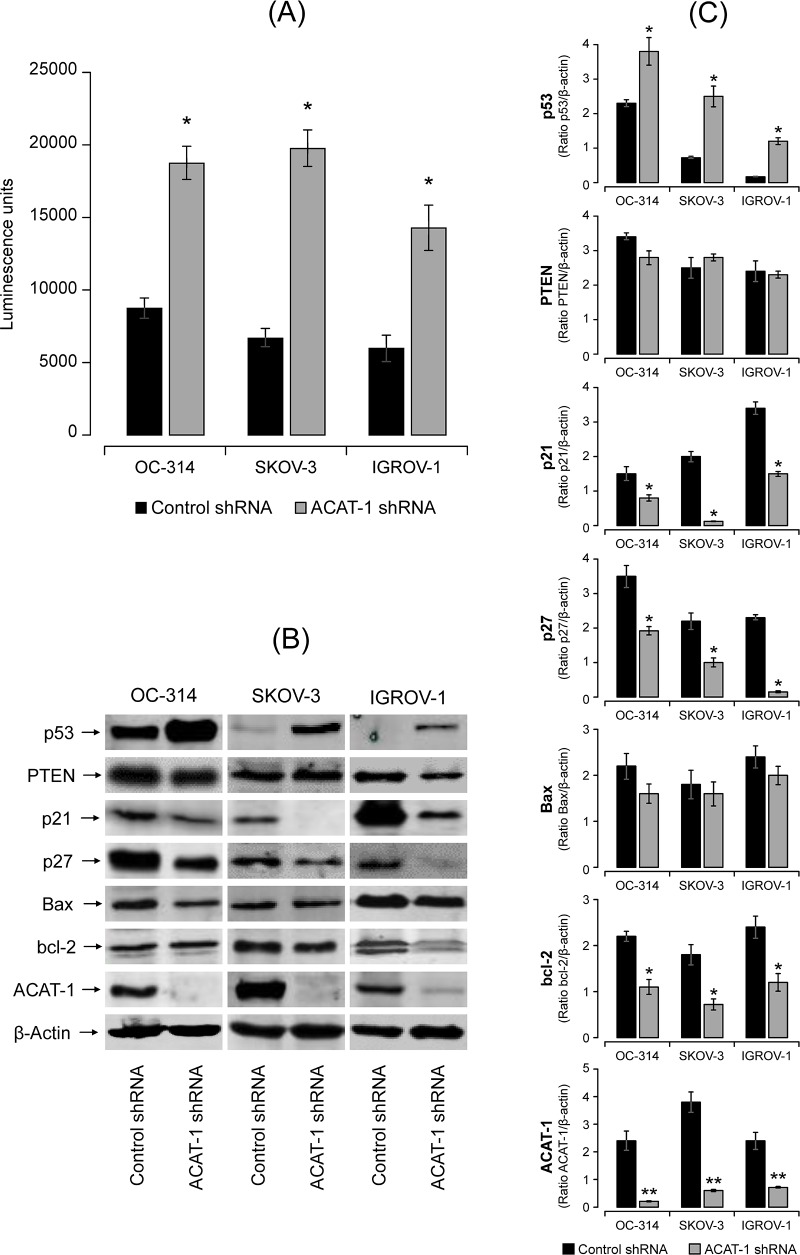
Effect of shRNA mediated ACAT-1 inhibition on apoptosis related proteins. **(A)** Effect of ACAT-1 inhibition on casapses3/7 activities. Data were expressed as mean ± SD of triplicate experiments. *p < 0.05, compared to scrambled control, Student’s t-test. **(B)** Western blot analysis of the expression of proteins in the cell lysates. The blots were probed with the respective primary antibodies. β-actin was used as the loading control. All blots were quantified using Odyssey software. **(C)** Western blot band intensity was quantified by scanning densitometry. Data are means ± SD. *P<0.05, **P<0.01.

To assess caspase3/7 activities, we utilized Promega’s Caspase-Glo3/7 assay kit that measures caspase-3 and -7 activities luminometrically. Fig 8 shows a significant increase (2–3 fold) in caspase-3/7 activities in ACAT-1 inhibited ovarian cancer cell lines compared to their respective scrambled controls. All the three cell lines showed similar results. Collectively, these results support the idea that ACAT-1 inhibition leads to enhanced apoptosis in ovarian cancer cell lines which may contribute to reduced cell proliferation.

#### ACAT-1 inhibition changes the expression of proteins associated with tumor suppression

In general, tumor suppression is associated with cell cycle arrest and apoptosis. In this study, we focused on the important tumor suppressor genes p53 and PTEN. Densitometric analysis of western blots showed expression of PTEN in all the three cell lines. There was no significant difference in PTEN expression between ACAT-1 inhibited cell lines of OC-314, SKOV-3 and IGROV-1 and their respective scrambled controls (Fig 8B and 8C). However, a significant increase in the expression of p53 was observed upon ACAT-1 inhibition in all three cell lines compared to their respective scrambled controls. This increase was irrespective of the p53 status of the cell lines (Fig 8B and 8C).

p53 restricts cellular growth by inducing apoptosis, cell cycle arrest or senescence. Therefore, we analyzed the expression levels cell cycle regulators p21, p27 and important bcl-2 family members, Bax and bcl-2. As shown in Fig 8B and 8C, western blot analysis showed significant decrease in expression levels of both p21 and p27 in ACAT-1 inhibited cell lines compared to their respective scrambled controls. Interestingly, slight but significant decrease in expression of anti-apoptotic protein bcl-2 was observed in ACAT-1 inhibited cell lines compared to their respective scrambled controls. However, no significant difference in Bax expression was observed.

#### ACAT-1 inhibition increased ROS production

**Fig 9:**
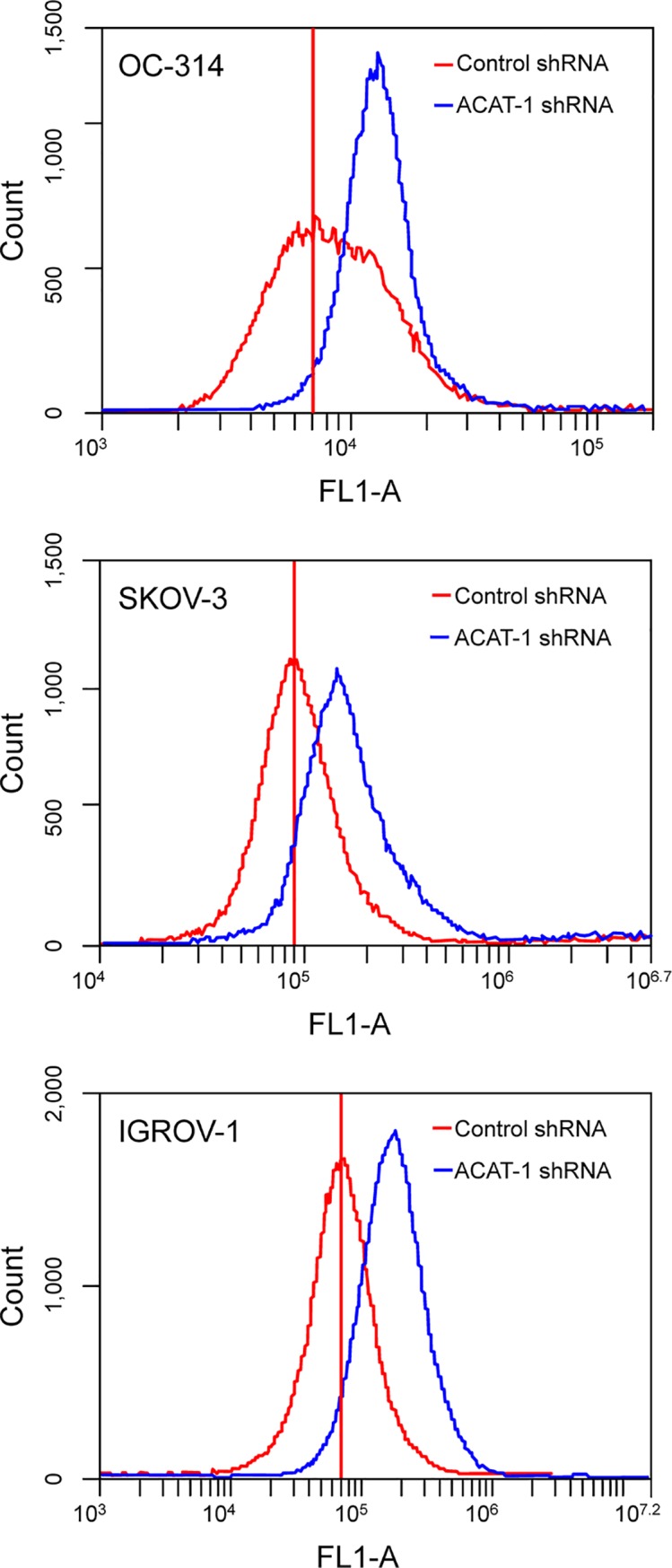
Effect of shRNA mediated ACAT-1 inhibition on reactive oxygen species (ROS). Detection of ROS was made by flow cytometry. Data were presented as relative-fluorescence intensity in a 2-dimensional FACS profile (standardized gating, 20,000 events). Enhanced ROS generation is observed as a shift the in peaks in ACAT-1 inhibited cell lines (blue) compared to scrambled controls (red). All experiments were performed in triplicate.

As ROS generation is commonly associated with mitochondrial depolarization and apoptosis [33], we investigated the effects of ACAT-1 inhibition on ROS levels. Flow cytometric analyses showed increased ROS levels in ACAT-1 inhibited cell lines as indicated by a shift in peaks, compared to their respective scrambled control cells. Similar results were observed in all three ovarian cancer cell lines (Fig 9).

### ACAT-1 inhibition and cisplatin sensitivity

**Fig 10:**
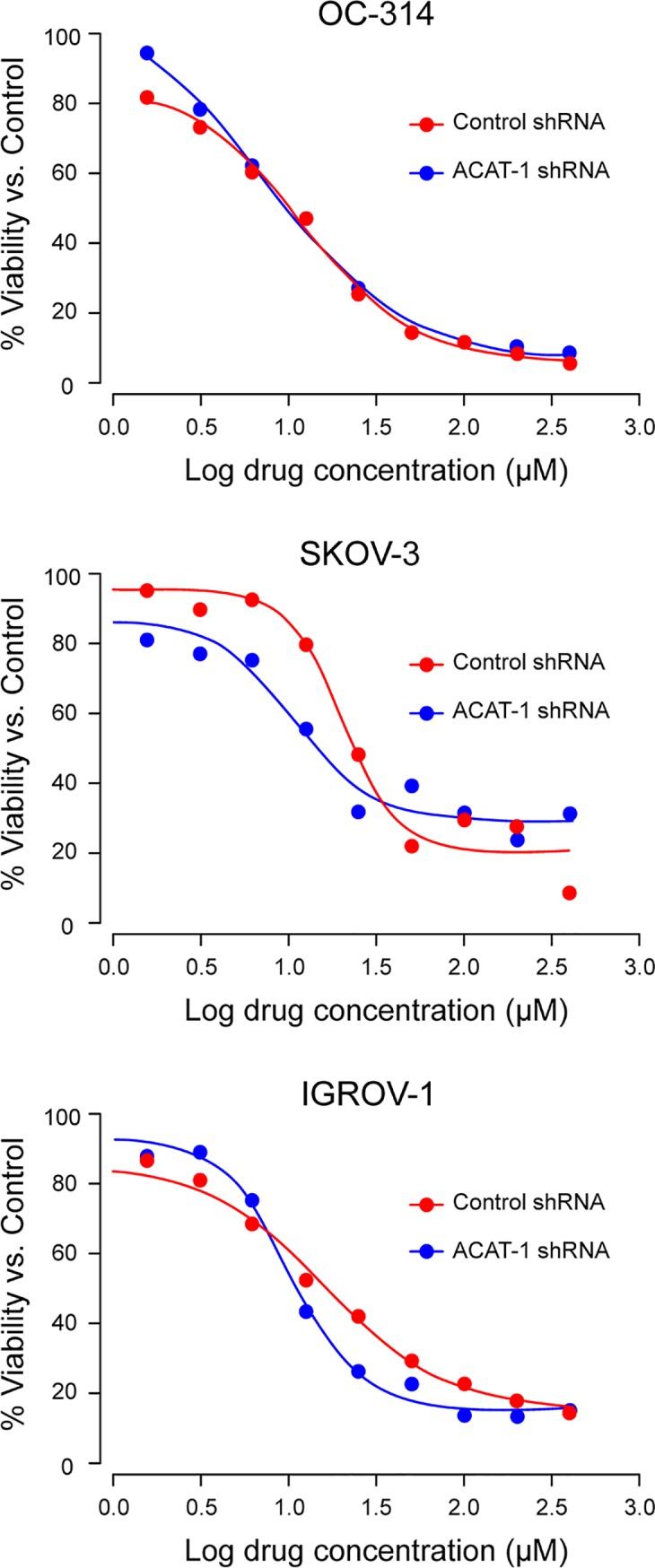
Cisplatin dose response curves. Cisplatin IC_50_ values were assessed and compared between ACAT-1 inhibited and scrambled controls. Cell viability was determined by PrestoBlue cell viability reagent. Dose response curves to calculate IC_50_ values were plotted using GraphPad Prism Software. Data were expressed as mean ± SD of triplicate experiments.

Cisplatin half maximal inhibitory concentration (IC_50_) values were assessed and compared between ACAT-1 inhibited and scrambled controls of OC-314, SKOV-3 and IGROV-1. Cells were treated with cisplatin at concentrations ranging from 0.78 μM to 400 μM for 48 hours. We observed a significant decrease in cisplatin IC_50_ values in ACAT-1 inhibited cell lines compared to their respective scrambled controls (Fig 10). ACAT-1 inhibited cell lines of OC-314, SKOV-3 and IGROV-1 cell lines showed cisplatin IC_50_ values of 11.88 ± 1.23 (vs. 7.736 ± 1.05 μM in the respective control), 20.28 ± 2.32 (vs. 10.77 ± 2.11 μM in the control) and 16.14 ± 2.45 (vs. 10.27 ± 1.64 μM in the control) respectively. Data were expressed as mean ± SD of triplicate experiments.

## Discussion

Cholesterol/lipid homeostasis is essential for the growth of mammalian cells. Differential regulation of lipidogenic and cholesterogenic pathways has been reported in ovarian cancer [34]. Among other metabolites, significant accumulation of CE due to increased ACAT-1 expression was reported in multiple cancers [11–14, 35, 36]. Suppression of CE accumulation via ACAT-1 inhibition appeared to significantly impair the tumor growth as observed in pancreatic cancer, prostate cancer and in glioma tissues [12, 27, 29]. These reports suggest that cholesterol esterification could be considered a novel potential target for suppression of cancer proliferation and metastasis. Additionally, the fact that inhibitors of ACAT-1 exhibited strong cancer-specific therapeutic potential in other cancers merits further investigation into gynecological cancers as well. In this study, we focused on the role of ACAT-1 and CE levels in ovarian cancer using a panel of ovarian cancer cell lines.

Similar to other cancers, this study revealed significant expression of ACAT-1 and CE in ovarian cancer cell lines compared to normal control cells. The cholesterol present in plasma membrane/lipid rafts was shown to have an essential role in cancer cell adhesion and migration [37]. Decreased aggressiveness of SKOV-3 cells was associated with deceased membrane fluidity and repressed cholesterol pathway [38]. In support of these reports, inhibition of CE/ACAT-1 either by avasimibe (data not shown) or by ACAT-1 specific shRNA, significantly reduced the migration, invasion and proliferation properties of the ACAT-1 inhibited cancer cell lines compared to their respective scrambled controls.

The anti-tumor effects observed upon ACAT-1 inhibition can be attributed to enhanced apoptosis as confirmed by elevated caspases and reduced mitochondrial membrane potential. Although we did not detect cell cycle arrest in any of the cell cycle phases, significant accumulation of cells in Sub-G1 (apoptotic) phase was observed. These results suggest that ACAT-1 inhibition causes the cells to undergo predominantly apoptosis rather than cell cycle arrest. Additionally, we observed increased generation of ROS in ACAT-1 inhibited cell lines compared to their respective scrambled controls. This is consistent with the fact that ROS generation is commonly associated with mitochondrial membrane depolarization [33, 39].

Excess CE may alter tumor cell signaling to promote tumor proliferation and survival [18–20] and depletion of CE suppresses cancer proliferation [12, 27, 29]. It still not clear which signaling molecules/pathways are predominantly altered by CE/ACAT-1 levels. Previous studies showed that loss of tumor suppressor, PTEN, and subsequent activation of PI3K/AKT/mTOR pathway may lead to accumulation of CE in advanced pancreatic cancer [14]. ACAT-1 inhibition was reported to lead to down-regulation of the caveolin-1/MAPK pathway, which contributed to reduced cancer aggressiveness [27]. *Ohmoto T et al*. showed that the selective ACAT-1 inhibitor K604 effectively suppressed the proliferation of U251-MG glioblastoma cells via reduced phosphorylation of Akt and ERK1/2 [23]. Collectively, these studies imply that ACAT-1 inhibition regulates cellular proliferation or cancer aggression via different pathways. In order to understand the molecular mechanism(s) underlying apoptosis or suppression of cancer cell proliferation upon ACAT-1/ CE inhibition in ovarian cancer cell lines, we focused on tumor suppressor genes associated with cell cycle and apoptosis.

Among various tumor suppressor proteins, p53 tumor-suppressor protein encoded by the TP53 is considered the “guardian of the genome”. TP53 also plays an important role in the regulation of cholesterol pathways [40]. Mutations in the TP53 gene are implicated in approximately 50 percent of cancers. In general, wild type p53 is considered a tumor suppressor, whereas mutated p53 is tumor promoter. In our study, western blot analysis of all three ACAT-1 inhibited cell lines showed significant increase in p53 expression compared to their respective scrambled controls. The three cancer cell lines tested in our study are reported to each exhibit a distinctive p53 status [41]. In general OC-314 cell lines are known to contain a mutated p53, IGROV-1 cells express wild-type p53 and the SKOV-3 cell line is p53-null. The p53 status of IGROV-1 and SKOV-3 cell lines is still controversial, as these cell lines have also been reported to contain mutated p53 [41]. ACAT-1 inhibition enhanced p53 expression and apoptosis in all three cell lines irrespective of their p53 status, suggesting p53 independent mechanism(s) underlying apoptosis. Still, we cannot rule out the significance of increased p53 levels upon ACAT-1 inhibition contributing to proliferation suppression.

Increased levels of p53 have been observed upon a variety of cellular stresses including genotoxic damages, oncogene activation and hypoxia [42–46]. p53 restricts cellular growth by inducing apoptosis, cell cycle arrest or senescence via transcription-dependent or transcription-independent mechanism(s) [47]. p53 is known to mediate apoptosis via trans-activation of pro-apoptotic protein Bax [48, 49] and trans-repression of anti-apoptotic protein bcl-2 [50, 51]. Similarly, p53 mediated trans-activation of cell cycle regulatory protein p21 is essential to induce cell cycle arrest [52, 53]. In our study, Bax expression did not change significantly upon ACAT-1 inhibition; however, a mild but significant decrease in anti-apoptotic protein Bcl-2 was observed. This decrease may be p53 mediated as p53 is known to induce apoptosis by inhibition of Bcl-2 protein levels, independent of Bax expression [54]. Interestingly, cell cycle regulatory proteins, p21 and p27, were down-regulated upon ACAT-1 inhibition. Thus, the lack of enhanced expression of p21 and Bax suggests that p53-mediated tumor suppression may be independent of its transcriptional function.

A number of p53 mutants were shown to display normal cell cycle arrest and apoptotic functions, while others lacked both activities. *Kokontis et al*. reported that wild type p53 induces cell cycle arrest in a p21 dependent manner, without apoptosis, whereas a p53 mutant (defective in trans-activation) induces apoptosis without cell cycle arrest [55]. He *et al*. reported that, similar to wild type p53, the pP53^gly (281)^ mutant was able to induce apoptosis despite the lack of p53-specific transactivation function [56]. In this study, we observed enhanced expression of p53 and apoptosis with lack of cell cycle arrest upon ACAT-1 inhibition irrespective of the cell line p53 status. Additionally, the observation of mitochondrial membrane potential loss along with caspases activation and generation of ROS may support the hypothesis that p53 (either wild type or mutant) may have induced apoptosis via the mitochondrial pathway through transcription-independent mechanisms. Since this is a preliminary report, further studies are needed to assess the contribution of p53 upon ACAT-1 inhibition in promoting tumor suppression.

Ovarian cancer recurrence has been mainly attributed to drug resistance even after initial successful response to platinum-based chemotherapy [3, 4]. Cancer drug resistance is attributed to metabolic reprogramming; therefore, targeting this aspect was considered beneficial to overcome drug resistance [57, 58]. Cholesterol metabolism was implicated in the development of resistance to tamoxifen in breast cancer [59]. Moreover, increased accumulation of CE was observed in gemcitabine-resistant pancreatic ductal adenocarcinoma cells [29]. These reports suggest that cholesterol esterification is likely to contribute to the development of drug-resistance through yet undetermined mechanisms. In our study, ACAT-1 inhibited cell lines of SKOV-3 and IGROV-1 displayed an increased sensitivity to cisplatin compared to their respective scrambled controls. Although we do not have evidence supporting a specific mechanism(s), others have reported that ACAT-1 inhibition and depletion of CE lead to inhibition of PI3K/Akt or caveolin pathway; thus, contributing to increased sensitivity to drugs [60, 61]. More research is needed to fully elucidate the mechanisms that link cholesterol metabolism and cancer drug resistance in ovarian cancer.

## Conclusions

In summary, our results show increased expression of ACAT-1 in ovarian cancer cell lines compared to the primary ovarian epithelial cells (normal controls); thus, confirming that ACAT-1 mediated CE accumulation is a cancer specific event. Inhibiting ACAT-1 and depleting CE levels seems to have an anti-tumor effect in terms of regulation of apoptosis, cell proliferation, migration and invasion properties in addition to enhancing the sensitivity to cisplatin. Therefore, therapeutic strategies to regulate ACAT-1/CE levels may prove beneficial for ovarian cancer treatments. It is essential to understand that multiple lipidogenic and cholesterogenic pathways are affected in ovarian cancer. Therefore, it is critical to target multiple lipid/cholesterol metabolism routes for effective ovarian cancer treatement as multiple feedback loops exist for compensating any single blockage.
